# Hemodynamic Signal Changes Accompanying Execution and Imagery of Swallowing in Patients with Dysphagia: A Multiple Single-Case Near-Infrared Spectroscopy Study

**DOI:** 10.3389/fneur.2015.00151

**Published:** 2015-07-06

**Authors:** Silvia Erika Kober, Günther Bauernfeind, Carina Woller, Magdalena Sampl, Peter Grieshofer, Christa Neuper, Guilherme Wood

**Affiliations:** ^1^Department of Psychology, University of Graz, Graz, Austria; ^2^BioTechMed Graz, Graz, Austria; ^3^Laboratory of Brain-Computer Interfaces, Institute for Knowledge Discovery, Graz University of Technology, Graz, Austria; ^4^Klinik Judendorf-Straßengel, Gratwein-Straßengel, Austria

**Keywords:** near-infrared spectroscopy, motor imagery, motor execution, swallowing, dysphagia, stroke

## Abstract

In the present multiple case study, we examined hemodynamic changes in the brain in response to motor execution (ME) and motor imagery (MI) of swallowing in dysphagia patients compared to healthy matched controls using near-infrared spectroscopy (NIRS). Two stroke patients with cerebral lesions in the right hemisphere, two stroke patients with lesions in the brainstem, and two neurologically healthy control subjects actively swallowed saliva (ME) and mentally imagined to swallow saliva (MI) in a randomized order while changes in concentration of oxygenated hemoglobin (oxy-Hb) and deoxygenated hemoglobin (deoxy-Hb) were assessed. In line with recent findings in healthy young adults, MI and ME of swallowing led to the strongest NIRS signal change in the inferior frontal gyrus in stroke patients as well as in healthy elderly. We found differences in the topographical distribution and time course of the hemodynamic response in dependence on lesion location. Dysphagia patients with lesions in the brainstem showed bilateral hemodynamic signal changes in the inferior frontal gyrus during active swallowing comparable to healthy controls. In contrast, dysphagia patients with cerebral lesions in the right hemisphere showed more unilateral activation patterns during swallowing. Furthermore, patients with cerebral lesions showed a prolonged time course of the hemodynamic response during MI and ME of swallowing compared to healthy controls and patients with brainstem lesions. Brain activation patterns associated with ME and MI of swallowing were largely comparable, especially for changes in deoxy-Hb. Hence, the present results provide new evidence regarding timing and topographical distribution of the hemodynamic response during ME and MI of swallowing in dysphagia patients and may have practical impact on future dysphagia treatment.

## Introduction

Dysphagia is a difficulty in swallowing that often occurs in neurological patients (1). Especially, dysphagia is a very common consequence of stroke, estimated to occur in up to 76% of patients with acute stroke (2). Dysphagia can have tremendous consequences, such as chest infection, malnutrition, prolonged hospital stay, slower rate of recovery, poorer rehabilitation potential, mortality, and reduced quality of life (3, 4). In the present study, we aimed at identifying brain correlates of active swallowing and mental imagination of swallowing in single stroke patients with dysphagia compared to healthy matched controls, which might be the basis for upcoming dysphagia rehabilitation (5).

Brain activation patterns associated with mental imagination of swallowing have been scarcely investigated. Based on numerous studies investigating brain correlates of motor imagery (MI) and motor execution (ME) of limb movements, it is well known that both tasks lead to similar brain activation patterns. Moreover, mental practice with MI can improve motor functions. Hence, MI strategies can be potentially used for motor rehabilitation (5–16). In a recent study performed in our lab, we investigated brain activation patterns associated with ME and mental imagery of swallowing in healthy young adults using near-infrared spectroscopy (NIRS). We found the strongest activation during both tasks in the inferior frontal gyrus bilaterally. Hence, this prior NIRS study provided first evidence that the hemodynamic response during ME and MI of swallowing is largely comparable in healthy people (5).

Brain activation patterns associated with active swallowing have been thoroughly investigated (17–37). Generally, swallowing is a complex and fundamental neuromuscular activity consisting of voluntary (oral phase) and involuntary (pharyngeal and esophageal phase) phases (38). Reflexive components of the swallowing process are controlled by swallowing centers in the brainstem (20). Acute focal brainstem infarct may produce dysphagia symptoms, especially affecting reflexive phases of the swallowing process, with little or no further neurocognitive deficit. About 62.5% of stroke patients with lesions in the brainstem, i.e., medulla oblongata or pons, aspirate (2). In contrast, volitional swallowing is represented in multiple regions of the cerebral cortex, such as the sensorimotor cortex, the lateral motor cortex, insula, the cerebellum, the superior temporal gyrus, middle and inferior frontal gyri, anterior cingulate cortex, and frontal operculum including the Broca’s area (21, 25, 38–40). Lesions in these cerebral regions impair voluntary swallowing movements, which may lead to aspiration, too (2, 41). On the basis of clinical and neuroimaging studies, especially unilateral hemispheric stroke can result in dysphagia (2, 42). A number of studies confirmed that about 40% of patients with unilateral hemispheric stroke may have swallowing difficulties (20).

These prior neuroimaging studies that investigated brain activation patterns underlying active swallowing were mainly based on functional magnetic resonance imaging (fMRI). In the present study, we used NIRS, which is a relatively new, non-invasive optical neuroimaging technique. NIRS measures concentration changes of oxygenated hemoglobin (oxy-Hb) and deoxygenated hemoglobin (deoxy-Hb) in the cerebral vessels based on their different absorption spectra for light in the near-infrared range (43). It is based on the same physiological signal as fMRI, since it measures the blood oxygenation level dependent (BOLD) effect in cortical areas (44). There is evidence that the oxy-Hb signal is the most sensitive indicator of changes in cerebral blood flow (CBF). The CBF is an indicator of cortical activation. In contrast, the direction of changes in deoxy-Hb is determined by the degree of changes in venous blood oxygenation and volume (45). Compared to fMRI, NIRS has low costs, is more flexible, and portable. Furthermore, NIRS has a higher temporal resolution than most commonly used fMRI scanners (46, 47) and also wireless, and portable instruments are available (47, 48). A further big advantage of NIRS over fMRI is that NIRS has a lower sensitivity to motion-artifacts (49, 50), allowing measurements of brain activation also in natural and realistic environments. However, with fMRI, the whole brain including sub-cortical areas can be recorded with a high spatial resolution (44). When using NIRS, only changes in hemodynamic responses a few centimeters (0.5–2 cm) below the surface of the head can be assessed (46). Summing up, the main advantages of NIRS are that it is not locally bounded to the installation site and can accommodate a higher degree of movement. Therefore, NIRS turned out to be an adequate neurophysiological method enabling the assessment of brain activation patterns of neurological patients in clinics or at the patients’ home (51).

The aim of the present multiple single-case study was to use NIRS to examine the cortical correlates of swallowing in patients with dysphagia. We focused on the comparability of brain activation patterns associated with saliva swallowing between dysphagia patients and healthy-matched controls. More precisely, we were interested in the time course and the topographical distribution of the hemodynamic signal change (oxy-Hb and deoxy-Hb) during swallowing in dysphagia patients compared to healthy controls. Special emphasis lay on the question whether there are differences in brain activation patterns during swallowing movements between dysphagia patients with lesions in the cerebral cortex and dysphagia patients with lesions in the brainstem. Since there is evidence that lesions in cerebral regions impair voluntary swallowing movements whereas lesions in the brainstem impair more involuntary phases of the swallowing process, we expected differences in cerebral activation patterns during swallowing in dependence on lesion location (2, 41). Furthermore, we wanted to determine the extent to which MI and ME of swallowing lead to comparable brain activation patterns in stroke patients, such as we have already observed in healthy young adults (5).

## Materials and Methods

### Participants

This study used a multiple case design (52, 53). ME and MI of swallowing were explored in four stroke patients with dysphagia matched for gender and age to two healthy control subjects. Patient details are summarized in Table 1. Recruitment of stroke patients and NIRS measurements were performed at the rehabilitation clinic Judendorf-Strassengel, Austria. Patients 12–19 and 12–23 had cerebral lesions in the right hemisphere, patients 01–07 and 01–28 had lesions in the brainstem. Assessment of swallowing abilities and determination of the degree of dysphagia were performed by speech and language therapists of the rehabilitation clinic Judendorf-Strassengel. Dysphagia was scored using the Bogenhauser Dysphagia Score BODS (54, 55). The BODS is a psychometric assessment of clinical dysphagia implying swallowing saliva as well as oral intake of food and liquids. It is a systematic rating of the functional severity of dysphagia. Content validity and inter-rater reliability were investigated. Psychometric data indicate that the BODS is an appropriate and reliable tool for the clinical evaluation of swallowing abilities of patients with neurogenic dysphagia (56). This study was approved by the local ethics committee of the University of Graz and conforms to the Declaration of Helsinki.

**Table 1:** **Patient description**.

Code	Degree of dysphagia^a^	Sex	Age	Handedness	ICD-10 diagnosis	Lesion location	Time since onset (days)	MMSE
12–19	4 (slight)	F	74	Rt	I63.9	Rt arteria cerebri media	254	25
12–23	5–6 (moderate)	F	68	Rt	I63.5	Rt arteria cerebri media	71	26
01–07	9 (moderate–severe)	M	78	Rt	I63.9	Lt medulla oblongata	71	29
01–28	11 (severe)	F	80	Rt	I63.5	Rt medulla oblongata	114	27

*Bt, bilateral; F, female; Lt, left; M, male; MMSE, mini-mental state examination; and Rt, right*.

*^a^Bogenhauser Dysphagia Score BODS (54, 55)*.

Selection of patients was based on the following criteria: in the first step and with the prior consent of the attending medical doctors or therapists, neurological patients who showed severe impairments in swallowing, with any site of brain lesion and motor deficit and with a time laps from the event of at least 4 weeks, were invited to participate in this investigation. Further inclusion and exclusion criteria are listed in Table 2.

**Table 2:** **Inclusion and exclusion criteria**.

Further inclusion criteria	Exclusion criteria
Good seeing and hearing (normal or corrected to normal) Understanding of the context and ability to give informed consent (the study conforms with the code of ethics of the World Medical Association, Declaration of Helsinki) Presence of a supportive person/environment	Other concomitant neurological disorders (e.g., Parkinson disease; visual-reflex epilepsy) Dementia (MMSE <24) Insufficient comprehension and communication ability (linguistic ability, reading, writing) Insufficient awareness, motivation and/or cooperation Psychiatric disorders Visual hemi-neglect extending to somatosensorial representations

Two neurologically healthy control subjects with no swallowing problems performed the swallowing task as well, one 67 years old man, and one 64 years old woman. The healthy control subjects were largely comparable to the stroke patients regarding age and gender.

### MI/ME task swallowing

The MI/ME task was performed in the rehabilitation clinic Judendorf-Strassengel. During the task, participants were sitting in front of a computer screen. The portable NIRS equipment was placed behind the participants. NIRS montage took about 5 min. In sum, all participants performed 10 trials of MI of swallowing and 10 trials of ME of swallowing in a random order. For the ME task, participants were instructed to successively swallow their own saliva two to three times, indicated by a sign on the computer screen. We did not use a water swallowing task in this study to prevent aspiration in dysphagia patients. For the MI task, participants were instructed to imagine to swallow saliva but not to execute any swallowing movement. Each trial lasted 15 s. Between task trials, a fixation cross appeared at the screen with a variable duration from 28 to 32 s. During these resting trials, participants were instructed to relax and to avoid swallowing as much as they could. Participants trained both tasks at the beginning of the experimental session to accustom oneself to the timing of the trials before starting the MI/ME tasks. The whole procedure including the NIRS montage took approximately 30 min.

### NIRS recordings and analysis

**Figure 1:**
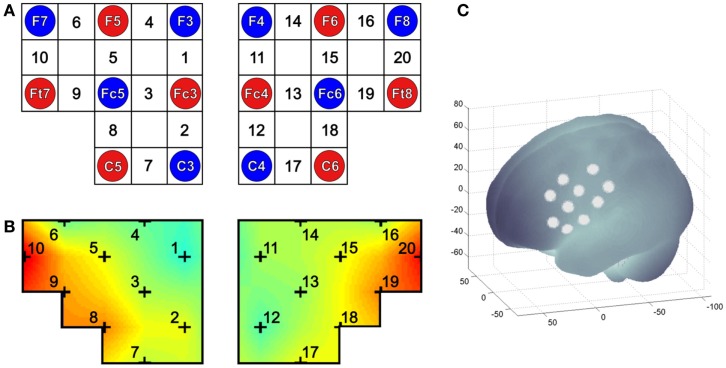
**(A)** Channel configuration of the optode probe set. The NIRS probe set of 20 channels was positioned over the right and left inferior frontal gyrus. Red and blue circles illustrate positions of NIRS sensors and detectors, respectively, and the white rectangles mark the measured 20 channels. **(B)** Example of a topographical map, in which the 20 NIRS channel locations are additionally marked. **(C)** Projections of 10 NIRS channel positions (white points) on the cortical surface over the left hemisphere. NIRS positions are overlaid on an MNI-152 compatible canonical brain that is optimized for NIRS analysis according to a procedure by Singh et al. (57).

To assess the relative concentration changes of oxy-Hb and deoxy-Hb during MI and ME of swallowing, measurements were performed on a continuous wave system, the NIRSport 88 system from NIRx Medical Technologies (Glen Head, NY, USA), consisting of 8 photo-detectors and 8 light emitters resulting in a total of 20 channels (see Figure 1). The sampling rate of the NIRS system was set to 10 Hz. The channel configuration of the NIRS probe set is illustrated in Figure 1. Based on the results of a recent study (5), channels were positioned over the inferior frontal gyrus bilaterally.

The MNI coordinates of the NIRS channels were assessed using ELPOS (zebris Medical GmbH), a system to determine 3D coordinates of EEG/NIRS electrode/optode positions with high accuracy based on the run time measurement of ultrasonic pulses. Afterwards, a coordinate-based system was used to retrieve brain labels from the 1988 Talairach Atlas, called the Talairach Daemon (58, 59). In Table 3, the anatomic labeling (Brodmann areas, Talairach Daemon) are listed for each NIRS channel.

**Table 3:** **Anatomic labeling of NIRS channel positions, Brodmann areas (Talairach daemon)**.

NIRS channel	Brodmann area	Description
1, 11	8	Includes frontal eye fields
2, 12	6	Pre-motor and supplementary motor cortex
3, 13	9	Dorsolateral prefrontal cortex
4, 14	46	Dorsolateral prefrontal cortex
5, 15	9, 45	Dorsolateral prefrontal cortex, Pars triangularis, which is part of the inferior frontal gyrus and Broca’s area
6, 16	45, 46	Pars triangularis, which is part of the inferior frontal gyrus and Broca’s area, Dorsolateral prefrontal cortex
7, 17	6	Pre-motor and supplementary motor cortex
8, 18	6	Pre-motor and supplementary motor cortex
9, 19	44	Pars opercularis, which is part of the inferior frontal gyrus and Broca’s area
10, 20	45, 47	Pars triangularis, which is part of the inferior frontal gyrus and Broca’s area, Pars orbitalis, which is part of the inferior frontal gyrus

For offline analyses of the NIRS signal, we investigated changes in oxy-Hb and deoxy-Hb separately. Before statistical analyses, the NIRS raw signal was preprocessed. Specifically, the raw data were corrected for artifacts (criterion for rejection: amplitude of Hb signal >±3 SD; visual inspection) and filtered with a 0.01 Hz high-pass filter to remove baseline drifts and a 0.90 Hz low-pass filter. The time courses of oxy-Hb and deoxy-Hb were averaged task-related separately for the MI and execution condition. Task-related concentration changes of oxy-Hb and deoxy-Hb were referred to a 5 s baseline interval prior to the MI/ME task (−5 to 0 s). Furthermore, oxy-Hb and deoxy-Hb were averaged for the time period around the peak activation during the MI/ME task (10–20 s after task onset).

## Results

### Topographical distribution

**Figure 2:**
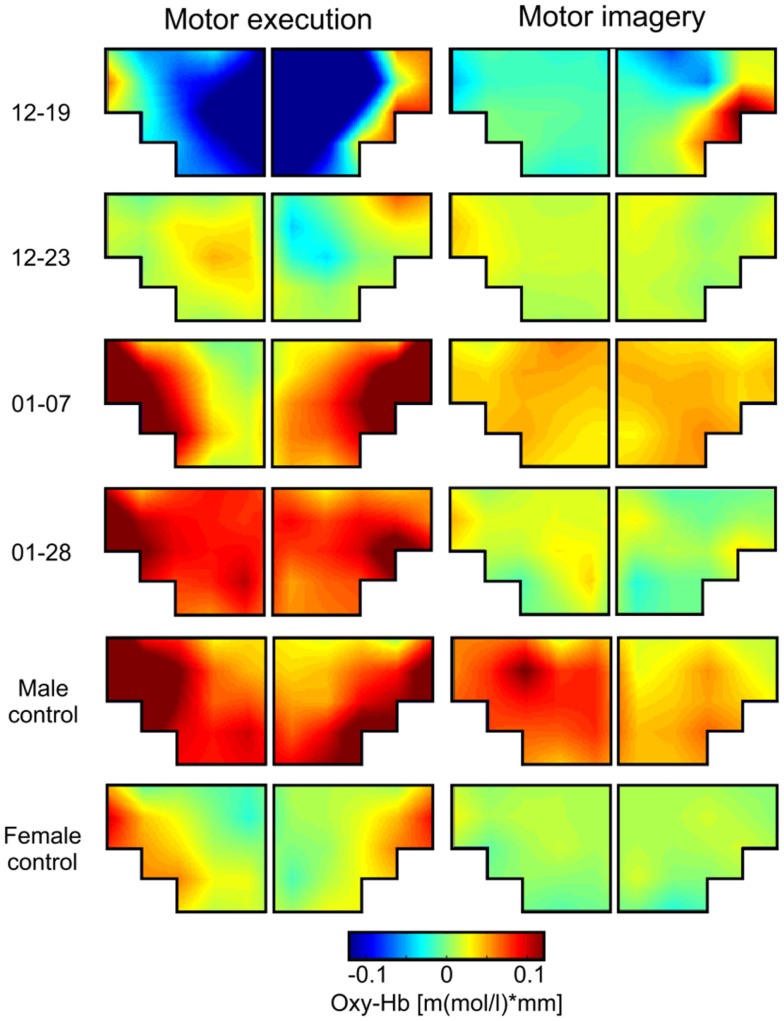
**Grand average topographical maps of oxy-Hb during motor execution (ME) and motor imagery (MI) of swallowing, presented separately for each participant [averaged time interval (second) 10–20 after task onset]**. The upper two panels show topographical maps of dysphagia patients with cerebral lesions (12–19 and 12–23), the middle two panels show topographical maps of dysphagia patients with lesions in the brainstem (01–07 and 01–28), and the lower two panels show maps of healthy controls.

**Figure 3:**
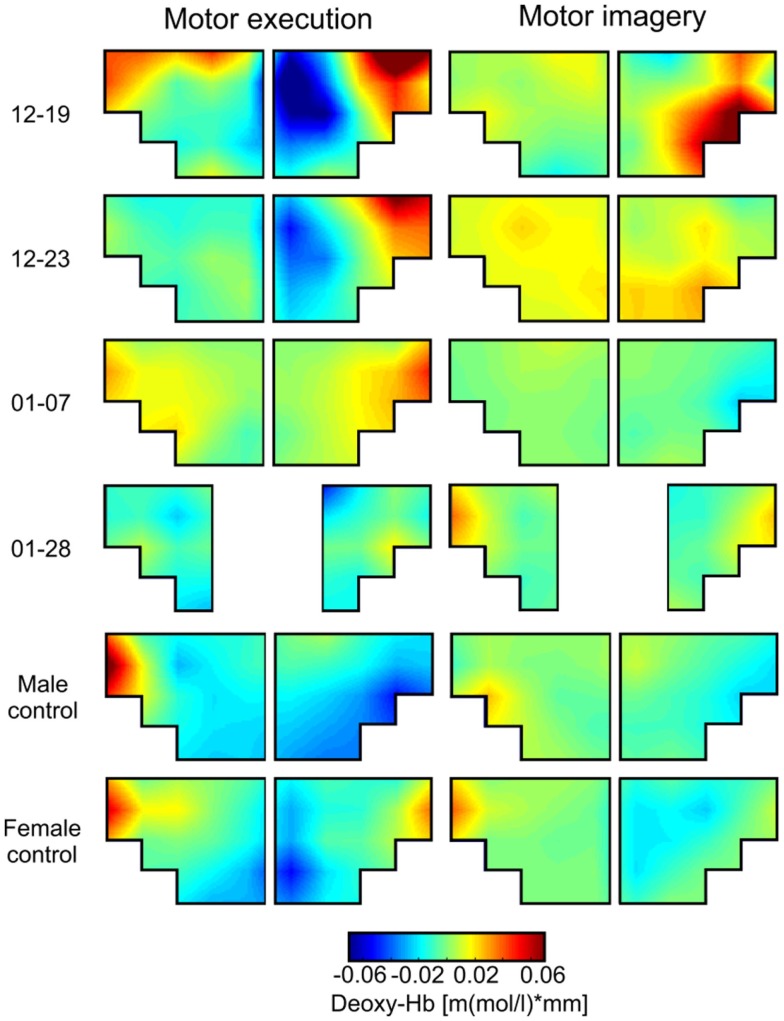
**Grand average topographical maps of deoxy-Hb during motor execution (ME) and motor imagery (MI) of swallowing, presented separately for each participant [averaged time interval (second) 10–20 after task onset]**. The upper two panels show topographical maps of dysphagia patients with cerebral lesions (12–19 and 12–23), the middle two panels show topographical maps of dysphagia patients with lesions in the brainstem (01–07 and 01–28), and the lower two panels show maps of healthy controls. Note that for patient 01–28 channel 1, 2, 11, and 12 had to be excluded because of bad signal quality.

First, we were interested in the topographical distribution of changes in oxy- and deoxy-Hb during ME and MI of swallowing in each single participant (Figures 2 and 3). To identify the NIRS channels that showed the strongest NIRS signal change (oxy- and deoxy-Hb) during ME/MI of swallowing compared to the baseline interval in each individual participant, we used the false discovery rate (FDR) method to control the proportion of false positives among the channels that are detected as significant (60).

This analysis revealed that during ME, relative concentration changes in oxy- (Figure 2, left panel) and deoxy-Hb (Figure 3, left panel) were strongest bilaterally over channels 9, 10, 19, and 20 in healthy controls as well as in patients 01–07 and 01–28 with lesions in the brainstem (*p* < FDR 0.10), except for the healthy male control subject, who showed the strongest deoxy-Hb increase only over left channels (9, 10) during ME. In contrast, patients 12–19 and 12–23 with cerebral lesions showed more unilateral activation patterns during active swallowing. In these two patients, relative concentration changes in oxy- and deoxy-Hb during ME of swallowing were strongest over the right hemisphere including channels 16, 19, and 20 (*p* < FDR 0.10).

During the MI task (Figures 2 and 3, right panel), patients 12–19 and 12–23 showed more unilateral activation patterns, too. Comparable to the ME task, changes in deoxy-Hb were strongest over the right hemisphere including channels 16, 18, 19 (*p* < FDR 0.10). For patients 12–19, changes in oxy-Hb during MI of swallowing were strongest over the right hemisphere including channel 18, 19, 20 (*p* < FDR 0.10), whereas for patients 12–23 changes in oxy-Hb during MI were strongest over the left hemisphere including channels 9 and 10 (*p* < FDR 0.10). For patients 01–07, no significant differences in oxy-/deoxy-Hb between NIRS channels could be observed during MI of swallowing. Patients 01–28 showed the strongest increase in oxy-Hb during MI over left hemispheric channels (channel 10), whereas changes in deoxy-Hb were more bilaterally distributed (channels 10, 20) (*p* < FDR 0.10). For the two healthy control subjects, the strongest activation changes during MI of swallowing were found over the left hemisphere including channel 5, 9, and 10 (*p* < FDR 0.10). As already observed for the ME task, topographical distribution patterns during MI of swallowing were largely comparable between healthy controls and patients with lesions in the brainstem. These results are summarized in Table 4.

**Table 4:** **Summary of the strongest relative concentration changes in oxy- and deoxy-Hb during ME and MI of swallowing over the inferior frontal gyrus (IFG)**.

	ME		MI	
	Oxy-Hb	Deoxy-Hb	Oxy-Hb	Deoxy-Hb
12–19	IFG right	IFG right	IFG right	IFG right
12–23	IFG right	IFG right	IFG left	IFG right
01–07	IFG bilateral	IFG bilateral	None	None
01–28	IFG bilateral	IFG bilateral	IFG left	IFG bilateral
Male control	IFG bilateral	IFG left	IFG left	IFG left
Female control	IFG bilateral	IFG bilateral	IFG left	IFG left

Brain activation patterns during MI and ME of swallowing were largely comparable, especially for changes in deoxy-Hb (see Figure 3). Both tasks led to the strongest NIRS signal change over the inferior frontal gyrus. Generally, the absolute amplitude of the NIRS signal was larger for active swallowing than for imagery of swallowing. Nevertheless, the topographical distribution of the strongest relative concentration change of oxy- and deoxy-Hb was comparable between both tasks.

### Time course of oxy- and deoxy-Hb during motor execution and imagery of swallowing

The topographical analysis revealed that changes in oxy- and deoxy-Hb during both tasks (MI and ME) were most pronounced in the inferior frontal gyrus including the Broca’s area (channels 6, 9, 10, 16, 19, 20). Based on the results of the FDR analysis, these channels were thus averaged to analyze differences in the time course of the NIRS signal during MI and ME of swallowing. Preliminary results revealed no hemispherical differences in the NIRS time course, therefore, channels of the right and left hemisphere were averaged for further analysis.

**Figure 4:**
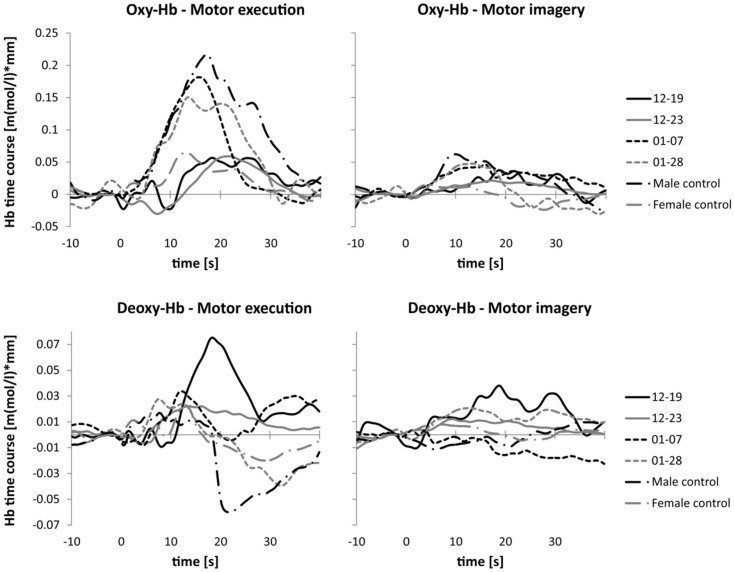
**Time course of the NIRS signal**. Mean activation changes in oxy- (upper panel) and deoxy-Hb (lower panel) in response to motor execution (left panel) and motor imagery (right panel), presented separately for each participant.

In Figure 4, the time course of oxy- and deoxy-Hb during MI and ME of swallowing is presented separately for each participant. The time courses of the NIRS signal of patients 01–07 and 01–28 with brainstem lesions were comparable to the time courses of the NIRS signal of the two healthy controls. The healthy controls and patients with brainstem lesions showed an earlier peak activation level in oxy- and deoxy-Hb during ME compared to patients 12–19 and 12–23. Hence, compared to the baseline interval, the NIRS signal increased faster in healthy controls and patients with brainstem lesions than in patients with cerebral lesions during ME of swallowing. The amplitudes of the NIRS signal of patients 01–07 and 01–28 were comparable to the absolute amplitude of the NIRS signal of the male control subject. The absolute amplitude level of oxy-Hb was generally lower during MI of swallowing than during ME in all participants. However, the time course of oxy-Hb during MI was comparable to the time course during ME. For the MI task, the peak of oxy-Hb seems to appear earlier in healthy controls and patients 01–07 and 01–28 compared to patients 12–19 and 12–23, too. No clear peak was detectable in deoxy-Hb during MI. In Table 5, the means and SE of the peak latency values for patients with cerebral lesions, patients with brainstem lesions, and healthy controls are presented separately for oxy- and deoxy-Hb and the ME and MI tasks.

**Table 5:** **Means and SE of peak latency values for patients with cerebral lesions, patients with brainstem lesions, and healthy controls, presented separately for oxy- and deoxy-Hb and the ME and MI tasks**.

	Mean (SE) peak latency (s)		
	Patients with cerebral lesions (12–19, 12–23)	Patients with brainstem lesions (01–07, 01–28)	Healthy controls
ME – oxy-Hb	23.25 (1.46)	14.54 (0.89)	16.01 (1.19)
MI – oxy-Hb	19.69 (0.84)	13.93 (0.78)	9.62 (1.54)
ME – deoxy-Hb	19.39 (0.89)	9.94 (2.21)	13.01 (0.67)
MI – deoxy-Hb	18.92 (1.85)	11.20 (1.45)	20.85 (3.77)

## Discussion

In the present study, we investigated hemodynamic signal changes accompanying ME and imagery of swallowing in stroke patients with dysphagia when compared to healthy controls using NIRS. We found differences in the topographical distribution and the time course of the hemodynamic response depending on lesion location. Dysphagia patients with lesions in the brainstem showed bilateral hemodynamic signal changes in the inferior frontal gyrus during active swallowing comparable to healthy controls. In contrast, dysphagia patients with cerebral lesions in the right hemisphere showed more unilateral activation patterns during swallowing. Furthermore, patients with cerebral lesions showed a prolonged time course of the hemodynamic response during MI and ME of swallowing compared to healthy controls and stroke patients with brainstem lesions. Brain activation patterns associated with ME and MI of swallowing were largely comparable, especially for changes in deoxy-Hb. In the following paragraphs, these results are discussed in more detail.

### Topographical distribution

In line with previous studies that investigated cortical correlates of swallowing (5, 18, 19, 21, 25, 61), we found an increased activation during swallowing in the inferior frontal gyrus including the Broca’s area. Generally, Broca’s area is considered to subserve motor speech production. Sensation of the mouth and pharynx was localized to BA 44, too (62), which indicates that the Broca’s area is also involved in the control of non-speech orofacial sensorimotor behaviors (21, 40). Furthermore, the increased activation around the Broca’s area during swallowing might be explained by the activation in deeper brain structures. For instance, the insula (i.e., BA 13) lies proximal to BA 44/45. Therefore, activation in BA 44 and 45 during the swallowing task might be caused by an increased activation in the insula. Various studies consistently report an involvement of the insula in the swallowing process (18, 19, 21, 40). Anatomical evidence shows that the insula receives afferent inputs from various brain regions involved in the swallowing process [e.g., BA 6, 1, 2, 3, 24, etc., for review, see Ref. (63)]. Furthermore, the inner side of BA 45 and the insular cortex are assumed to represent a primary gustatory cortex (21, 25, 40, 64–67). Damage to the insula and inner side of the operculum (BA 44, 45) often leads to dysphagia in humans underlining the importance of these brain regions for the swallowing process (2, 26, 68). However, activation levels of deeper brain structures, such as the insular cortex (BA 13), cannot be assessed by using NIRS, since NIRS measures change in hemodynamic responses only a few centimeters (0.5–2 cm) from the surface of the head (46).

Regarding lateralization effects during swallowing, we found a more bilateral activation in healthy controls and patients with lesions in the brainstem. Prior studies report on heterogeneous results. Some studies found that cortical activation patterns during swallowing were mainly bilateral (5, 18), whereas other studies found strong degrees of interhemispheric asymmetry (19, 40). Lateralization may also change with age. As people age, the cortical hemispheric control of swallowing seems to start becoming more bilateral (69). Participants of the present study were over 60 years old. Probably, the advanced age of the present cases might be a reason for the more bilateral activation observed during swallowing.

In contrast, stroke patients with lesions in the right ACM showed a more unilateral activation during swallowing. They showed a stronger increase in oxy- and deoxy-Hb during ME of swallowing in the right hemisphere, which was the affected hemisphere. The healthy left hemisphere was less active during swallowing. Generally, natural recovery of swallowing after stroke is associated with increased cerebral activation in the unaffected hemisphere (40). For instance, using transcranial magnetic stimulation (TCMS), Hamdy et al. (17) investigated functional changes in swallowing-related brain areas in stroke patients with lesions in the right hemisphere showing dysphagia symptoms 3 months after stroke. Comparable to the present findings in stroke patients with lesions in the right hemisphere, they found that dysphagia was associated with a smaller cortical activation in the intact hemisphere. The cortical activation in the unaffected hemisphere increased in size with recovery of swallowing (17). Furthermore, there are areas of hypometabolism at sites not directly affected by the stroke. This reduction in metabolism in unaffected areas may explain the representation and subsequent recovery as the metabolic state returns to baseline (2). Khedr et al. (70) confirmed that both severity of stroke and neuroplasticity of the unaffected hemisphere have implications for the development of dysphagia (70). In summary, natural recovery of dysphagia after a brain lesion, e.g., caused by a stroke, seems to be due to an improvement in the functional control of swallowing in the unaffected hemisphere. Hence, swallowing might recover by improving the output from the healthy side of the brain (2, 71, 72). Our findings are in line with these previous studies, since we found a lower cortical activation level during swallowing in the healthy hemisphere compared to the affected hemisphere in patients 12–19 and 12–23.

### Time course of oxy-Hb during motor execution and imagery of swallowing

In line with previous NIRS studies examining healthy young participants, the mean peak latency of oxy-Hb during ME of swallowing was around 15 s after task onset in healthy elderly controls as well as in patients 01–07 and 01–28, who had lesions in the brainstem (5, 61). A decrease in deoxy-Hb after ME around 10–15 s, which can be seen in the healthy controls and in patients with brainstem lesions, is also comparable to the deoxy-Hb time course of healthy young participants (5). Furthermore, the time course of the NIRS signal during active swallowing is comparable to the time course of the BOLD signal found in prior fMRI studies investigating neuronal correlates of swallowing (18, 21).

During MI of swallowing, oxy- and deoxy-Hb increased in the inferior frontal gyrus in healthy controls as well as in patients with lesions in the brainstem. An increase in deoxy-Hb during MI of swallowing was also found in healthy young adults (5). However, in healthy elderly participants, we found an increase in oxy-Hb during MI, whereas in a previous study on younger participants, oxy-Hb decreased during MI of swallowing (5). A decrease in oxy-Hb during MI of swallowing compared to a rest period was explained by possible motor inhibition mechanisms (5, 73). Generally, MI contains on the one hand ME programs for muscle contractions, but, on the other hand, these programs might be blocked at some level of the motor system by inhibitory mechanisms, which might in turn lead to a decreased activation level in swallowing-related brain areas (7, 74). In the present pilot study, we did not have the opportunity to assess the activity of muscles involved in swallowing, e.g., suprahyoid muscles, by using, for instance, the electromyogram EMG (75). Possible muscle activity during MI of swallowing was controlled by visual inspection. However, we cannot exclude that participants produced small movements during MI of swallowing, which could not be detected by eye. An incomplete inhibition of the motor command might explain that MI and ME of swallowing lead to an increase in oxy-Hb during both tasks (7). Prior studies in neurological patients also showed diminished inhibitory control, which led to involuntarily movements while imaging motor actions (7).

Patients with cerebral lesions showed a prolonged time course of the NIRS signal compared to healthy controls and patients with brainstem lesions. This prolongation probably reflects problems in initiating the voluntary swallowing process in these patients, or afferent/efferent feedback loops from the cortex to the periphery may work slower and lead to this sustained cerebral response (18, 21).

### MI vs. ME

Generally, both ME and MI tasks lead to the strongest NIRS signal change over the inferior frontal gyrus in all participants. Hence, MI and ME of swallowing lead to comparable brain activation patterns in stroke patients. Especially, changes in deoxy-Hb were most comparable between the ME and MI tasks. This result is in line with a previous NIRS study in healthy young adults. In that study, a stronger congruency was found in deoxy-Hb changes between ME and MI of swallowing than in oxy-Hb changes (5).

For oxy-Hb, the relative concentration change between resting period and task period was overall stronger for the ME than for the MI task. However, ME of any movement task generally leads to stronger brain activation patterns compared to mental imagery of the corresponding movements (5, 14, 16).

### Impact

The results of the present study may have practical impact on the future treatment of dysphagia. MI can be potentially used for motor rehabilitation since MI activates brain areas comparable to those during ME (5, 6, 16, 76). There is evidence that mental imagery of a specific motor task leads to a neuronal reorganization and consequently to improvements in motor functions (22, 77–81). However, prior MI studies focused on hand and food movements (14–16, 78). MI of swallowing movements might have comparable effects and might harness plasticity of the brain to improve the recovery of swallowing functions after brain injury (6, 82). However, this is a matter of future investigations.

### Limitations

Generally, multiple case studies enable the researcher to explore differences within and between cases. The goal is to replicate findings across cases, as we could show for patients with cerebral lesions and brainstem lesions. It is necessary that the cases are chosen carefully so that one can predict similar results across cases, or predict contrasting results based on a theory (52). We tried to select our cases carefully with comparable brain lesion sites, time since onset, cognitive abilities (MMSE), and degree of dysphagia (53). Case studies are valuable only if the case definition is precise and often raise issues of patient selection and comparability with other populations (83). However, they offer a practical and more rapid method of neuroscientific investigations. We believe that this design is appropriate for the initial phase of the scientific evaluation of cortical correlates of MI and ME of swallowing in dysphagia patients. However, the present results are based on a small sample of stroke patients. Hence, our results are more hypothesis generating and future studies with larger samples are needed to demonstrate the potential usefulness of mental imagery of swallowing for the treatment of dysphagia.

## Conclusion

In the present study, we revealed changes in hemodynamic responses accompanying MI and ME of swallowing in dysphagia patients using a relatively new imaging technique, the NIRS. Comparable to findings in healthy participants, we found the strongest hemodynamic changes in the inferior frontal gyrus during both ME and MI of swallowing in dysphagia patients. Furthermore, we could provide first evidence that localization of hemodynamic correlates of swallowing differ in dependence on brain lesion location. Hence, the present results provide new evidence concerning timing and topographical distribution of the hemodynamic response during ME and MI of swallowing in dysphagia patients, which might have practical impact on future dysphagia treatment. To the best of our knowledge, this is the first study investigating cortical correlates of MI and ME of swallowing in stroke patients with dysphagia symptoms.

## Conflict of Interest Statement

The authors declare that the research was conducted in the absence of any commercial or financial relationships that could be construed as a potential conflict of interest.
